# Should autism be considered a canary bird telling that *Homo sapiens* may be on its way to extinction?

**DOI:** 10.3402/mehd.v23i0.19008

**Published:** 2012-08-24

**Authors:** Olav Albert Christophersen

**Affiliations:** Pensioned Norwegian state stipendiate

**Keywords:** autism, germ-cell mutation rate, docosahexaenoic acid (DHA) deficiency, xenobiotics, Eigen error threshold, species extinction

## Abstract

There has been a dramatic enhancement of the reported incidence of autism in different parts of the world over the last 30 years. This can apparently not be explained only as a result of improved diagnosis and reporting, but may also reflect a real change. The causes of this change are unknown, but if we shall follow T.C. Chamberlin's principle of multiple working hypotheses, we need to take into consideration the possibility that it partly may reflect an enhancement of the average frequency of responsible alleles in large populations. If this hypothesis is correct, it means that the average germline mutation rate must now be much higher in the populations concerned, compared with the natural mutation rate in hominid ancestors before the agricultural and industrial revolutions. This is compatible with the high prevalence of impaired human semen quality in several countries and also with what is known about high levels of total exposure to several different unnatural chemical mutagens, plus some natural ones at unnaturally high levels. Moreover, dietary deficiency conditions that may lead to enhancement of mutation rates are also very widespread, affecting billions of people. However, the natural mutation rate in hominids has been found to be so high that there is apparently no tolerance for further enhancement of the germline mutation rate before the Eigen error threshold will be exceeded and our species will go extinct because of mutational meltdown. This threat, if real, should be considered far more serious than any disease causing the death only of individual patients. It should therefore be considered the first and highest priority of the best biomedical scientists in the world, of research-funding agencies and of all medical doctors to try to stop the express train carrying all humankind as passengers on board before it arrives at the end station of our civilization.

Autism is an etiologically heterogeneous group of metabolic disturbances that affect various organs, including the gut with its associated microflora as well as the brain, and lead to severe disturbance of cognitive development. Certain biochemical lesions that undoubtedly must have a genetic cause can be identified in part of the patient population, but not in all autistic patients, and may often be utilised for individualized therapy by avoiding food intolerances or compensating for the genetic biochemical lesion(s). These observations combined with the results of twin studies leave no doubt that the causes of autism and related disorders partly must be genetic, but most likely involving several different genes since the concordance is much higher in one-egged than two-egged twins (1) and the genes involved seem to be different in different patients. Autism is therefore etiologically not one single disease, but rather a mixed bag of several different diseases that have similar symptoms, although the precise combination of genetic and other causes is different in most patients with exception of one-egged twins and other close relatives. This increases the total number of cases compared to genetic disorders that etiologically can be more precisely defined (involving the same genes in all patients, though not necessarily the same DNA bases), which makes it easier to detect epidemiologically that the incidence is significantly different in different populations or that a significant change in total incidence has occurred over a certain period of time in the same population.

It can be inferred from the twin studies (comparing the concordance in one-egged and two-egged twins) that a doubling of the frequency of responsible alleles in the entire population will cause the incidence of autism to rise by a factor of 16, while tripling of this frequency will cause the incidence of autism to rise by a factor of 81. In the United States, this would mean that more than 80% of all children born will be autists and in South Korea close to 100%.

The genetic component in the etiology of autism is heavy enough that it is difficult to explain the dramatic enhancement of the reported incidence of autism and related disorders over only some few decades that has been reported in different parts of the world, including the United States (2–5), either by better diagnosis (with more cases now being discovered and reported) or by historical changes in environmental/life-style causal factors alone. Improved diagnosis and reporting may, however, explain some of the historical change in the reported incidence of autism that has taken place over the last 30 years, e.g. in California (where thorough epidemiological studies have been carried out), but apparently not all. It is also difficult to pinpoint environmental/lifestyle factors that might explain the dramatic enhancement in the reported incidence of autism by changes in non-genetic causes. General environmental pollution (e.g. lead emission from cars (6), DDT) was worse in the United States before 1975 than over the last 30 years, as was most likely also the combined burden of Pb, Sn, Hg and Ag from food, drinking water and dental amalgam fillings, while Cd ingestion has perhaps not been reduced to a similar extent (7). New long-lived pollutants (*e.g*. bromine compounds used as flame retarders) have, however, come instead of some of the old ones (8), and it can not a priori be excluded that some of them might play a role as causal factors in autism. There has, moreover, also been important changes in the use of drugs, and it can not a priori be excluded that some of the ‘new’ drugs (that are used more abundantly now than before 1985) might play a role as etiological factors, and not only as a consequence of the mutagenic effects of some of the substances concerned (e.g. paracetamol being mutagenic against mitochondrial DNA in the brain (9)).

If we shall follow T.C. Chamberlin's principle of multiple working hypotheses (10, 11), we need nevertheless to consider the possibility that it might also be the genetic causes of autism, i.e. the frequency of responsible alleles in the affected populations, that could have changed significantly over a period of only 30 years. If this is correct, it means that the average germ-line mutation rate in large populations, such as the entire population of the United States, must now be much higher than the natural mutation rate in humans and chimpanzees. It is therefore of utmost importance, may be even for the survival of our species, to find out if this hypothesis is true or not.

We need to ask if there are other observations suggesting that a substantial enhancement in the mutation rate may have happened in human germline cells, compared to the natural situation before the agricultural and industrial revolutions. The answer to this question is affirmative, since it is known that the total exposure in large human populations to a vast array of chemical mutagens (*i.a*. from tobacco, alcohol abuse, mutagenic drugs and mutagenic pesticides and food additives) is very high, and it is also known that impaired human semen quality is an enormous problem in several countries, including Norway and Denmark (12, 13). It has been reported that impaired semen quality very commonly is associated with enhanced oxidative stress (14–16), which must be expected to cause enhanced mutation rates (16–18) both in mitochondrial and nuclear germ-cell DNA. When too many mutations have accumulated in mitochondrial DNA (19, 20), this will lead to reduced synthesis of mitochondrial RNAs and of mitochondrially coded proteins that are needed as components of respiratory chain enzymes. This leads to impairment of ATP production (20) that might directly explain impaired sperm motility (because the ‘swimming motor’ of the spermatozoa becomes too weak), but also enhanced mitochondrial ROS production (20) that will enhance the probability of mitochondrially induced (21, 22) apoptosis before final differentiation of the male germ cells into mature spermatozoa, thus offering a plausible explanation for the reduced number of spermatozoa that is also commonly observed in infertile men (cf. also 23, 24).

Enhancement of the rate of mitochondrial ROS production because of too much mitochondrial DNA mutations may, however, lead also to enhanced attack on nuclear DNA by ROS (25) and mutagenic products of lipid peroxidation (26–29). It is important to notice that mitochondrial DNA aging is in principle a self-accelerating process in all cell types, since enhancement of the rate of mitochondrial ROS production because of the accumulation of mutations in mitochondrial DNA will itself lead to enhancement of the rate of attack on mitochondrial DNA by ROS (30), peroxynitrite (31) and products of lipid peroxidation. The rate of mitochondrial ROS production in male germ cells may therefore be expected to follow a quasi-exponential curve as a function of paternal age, at the same time as men now at the average wait longer before they become fathers than was common in traditional societies before the industrial revolution.

If we ask for possible causes of enhanced mutation rates in male germ-cell mitochondrial DNA, we have to consider not only the entire array of unnatural chemical mutagens (or natural ones at unnaturally high levels) to which large human populations are now exposed, but also dietary deficiency conditions that are mutagenic, e.g. because of impairment or disturbance of important DNA repair mechanisms. Among dietary deficiencies that very likely may have mutagenic effects in male germ cells, deficiency of docosahexaenoic acid (DHA) (9, 32–36) is probably most widespread (since it is very common both in rich and poor countries) and perhaps also most serious in terms of total damage to germ cell nuclear DNA in the entire world population. It happens as a consequence of unnaturally high omega-6/omega-3 fatty acid ratios both in commonly eaten animal foods (such as pork meat, poultry meat and beef meat from animals fed on maize instead of grass) and edible fats and oils (9). It is possible that DHA deficiency leads to impaired fluidity of the inner mitochondrial membrane (9). The rate of lateral diffusion of molecules participating in the respiratory chain will therefore be reduced, which leads to enhanced Ohmian resistance for electron transport through the respiratory chain and enhancement of the ratio between rates of mitochondrial ROS and ATP production (9).

Another likely cause of enhanced germ-cell mutation rates is toxic heavy metals inhibiting thioredoxin reductase both in male and female germ cells. Thioredoxin reductase is especially vulnerable to toxic heavy metals because it can form chelates where the toxic metal atom is simultaneously coordinated both to selenium and sulphur (in selenocysteyl and cysteyl groups sitting in vicinal positions) (37). Thioredoxin is used as reducing cofactor by 2-Cys peroxiredoxins, which scavenge H_2_O_2_, organic hydroperoxides and peroxynitrite (38–41) and have been reported to be present both in male (42–47) and female (48–51) germ cells, at least in non-human species in the case of females. Thioredoxin is also used by ribonucleotide reductase as one of two alternative reducing substrates (52). Alternatively, glutaredoxin can also be used as a reducing substrate by ribonucleotide reductase (52), with glutaredoxin reutilisation being dependent on reduced glutathione (GSH) (52, 53), while ribonucleotide reductase itself is an iron protein (54). Too much depletion of deoxynucleotides because of impaired electron transport to ribonucleotide reductase (especially when inhibition of thioredoxin reductase is combined with GSH depletion) may, of course, be expected to have important consequences not only for the rate of DNA synthesis in rapidly replicating cells (including leukocytes, enterocytes and male germ cells), but also for all DNA repair processes in the mitochondria as well as in the nucleus. There is therefore good reason to suspect that several toxic heavy metals by inhibiting thioredoxin reductase may lead to simultaneous impairment both of antioxidant/antinitrative defence and DNA repair in the germ cells both of men and women, with this genotoxic effect of heavy metals being enhanced if GSH is simultaneously depleted. In the male germ cells, it can also be expected that there will be a synergistic interaction between such heavy metal-induced disturbances and enhancement of the rate of mitochondrial ROS production because of DHA deficiency. It might be speculated, very tentatively, that such factors might help to explain the very high incidence of autism now found in South Korea (55), with Cd perhaps being one of the major culprits. The average dietary Cd intake is very high in Japan (56, 57), compared to the Scandinavian countries, but the Japanese are protected against Cd toxicity by also eating lots of Se from fish and other seafood (58). Perhaps the average dietary intake of Cd might also be high in South Korea (e.g. from Cd-containing fertilizers used on the rice fields), but without an equally high average intake of the antidote (59–61) Se as in Japan.

Other dietary deficiencies causing impairment either of antioxidative defence or DNA repair mechanisms, at least in somatic cell types (e.g. sulphur amino acid, Se, Zn, Fe, folate, vitamin B_12_ and niacin/tryptophan deficiencies), are very widespread when considering the entire world population, but some of them are much more common among poor people living in poor countries than they are in the more affluent parts of the world (62–69). Vitamin B_12_ and folate deficiencies can lead to uracil misincorporation into the DNA molecule instead of thymine during DNA synthesis or repair (70–75). This error can be repaired (76), but when TTP is deficient, misrepair is common, leading to DNA double-strand break (71) and chromosomal abnormalities, such as micronucleus formation (77). Zinc deficiency may have a similar effect because thymidine kinase (which is needed for thymidine reutilization by the salvage pathway) is a Zn-dependent enzyme vulnerable to Zn deficiency (78). The most recent estimate for the total number of people in the world suffering from Zn deficiency is close to 2 billions (79), while the total number of people suffering from anemia because of Fe deficiency is more than 1.6 billions (80). It is possible, though, that human germline cells may be better homeostatically protected than most somatic cell types against some of the dietary deficiencies concerned, but this question has never been adequately studied except for Se in the testicles (where the capacity for homeostatic regulation in some non-human species is better than in most other organs (81–83), but not complete either in animals or humans (59, 84–86)). We do not know, for instance, enough about what consequences vitamin B_12_ deficiency, Fe deficiency, Zn deficiency or GSH depletion may have for the DNA molecules of the germline cells either in foetuses and babies or in adult men or women.

Metabolic degradation of alcohol is associated with production of several mutagenic species, including acetaldehyde (87, 88), 1-hydroxyethyl radical (89, 90) and ROS (91, 92). Alcohol abuse has also important indirect effects by leading to enhanced degradation or excretion of several nutrients needed for normal antioxidant defence and/or DNA repair (9, 93–100) – which is nevertheless more important for people living on qualitatively poor diets than when the quality of the diet is very good. It is a reasonable speculation that alcohol abuse may be important not only as a cause of cancer (especially in the liver), but also as one of the important causes of enhanced germline cell mutation rates both in men and women, especially in countries with high alcohol abuse, such as Russia, and when alcohol abuse is combined with poverty and poor diets. But we do not have enough research data either from epidemiological or biochemical studies that this speculation should be regarded as more than a plausible working hypothesis.

We need also to take into consideration the possibility of conversion of xenobiotics other than alcohol (e.g. various drugs) into mutagenic metabolites by drug-metabolizing enzymes (especially various forms of cytochrome P_450_) that might be expressed either in male or female germ cells. However, it has apparently never been systematically studied how many of the well-known drug-metabolizing enzymes can be expressed in human germ cells either in men or women. It is therefore in most cases not possible to know, even when it is known that a drug is converted by some particular CYP into some mutagenic metabolite, if this will also happen in human oocytes or male germ cells, as long as it is not known if the CYP concerned is expressed in human germ cells or not. Epidemiological studies of the association of drug consumption with germline cell mutations are also few. However, it was found in a study from Iran that there was an association between consumption of selective serotonin reuptake inhibitors (SSRIs) and male germ cell DNA damage (101). If this observation can be confirmed by other scientists, it suggests that the testicles may contain some CYP capable of converting SSRIs into mutagenic metabolites, and there might be good reason for suspicion that the same might happen with some of the other commonly used drugs as well. The possible identity of this CYP, being presumably expressed in human male germ cells before their terminal maturation into spermatozoa is, however, still completely unknown.

In a study from California (102), prenatal exposure to antidepressant medications was reported for 20 case children (6.7%) and 50 control children (3.3%). In adjusted logistic regression models, it was found that treatment of the mother with SSRIs during the year before delivery was associated with a 2-fold increased risk of ASD in the child (adjusted odds ratio, 2.2 [95% confidence interval, 1.2-4.3]), with the strongest effect being associated with treatment during the first trimester (adjusted odds ratio, 3.8 [95% confidence interval, 1.8-7.8]) (102). No increase in risk was found for mothers with a history of mental health treatment in the absence of prenatal exposure to selective serotonin reuptake inhibitors (102). These data are compatible with a hypothesis that SSRIs may be mutagenic also in oocytes, egg cells or foetuses, similarly as has been reported for male germ cells (101), and that this may enhance the risk of ASD in the child. It is not unreasonable, if this hypothesis is found to be correct, that the enzyme(s) converting SSRIs into mutagenic (redox-cycling?) metabolites might be the same in both sexes.

The normal mutation rate in nuclear DNA is much higher in male germ cells than in oocytes because of the high growth rates of the former, whereas the oocytes of adult women are dormant until shortly before their final differentiation into mature egg cells (103). This may presumably also make the male germ cells more vulnerable to most of the unnatural causes of enhanced mutagenesis that are common in modern societies, with the possible exception of drugs that are used exclusively by women (such as contraceptive pills containing estrogen).

It is possible, however, that the human ovaries may contain the cytochrome P_450_s called CYP1A1 and CYP1B1 (as has been found in other mammalian species (104–106), but never seems to have been adequately studied in human oocytes or egg cells, although CYP1B1 has been found in other cell types in human ovaries (107, 108)), which are enzymes converting estrogens into catecholestrogens (109, 110). The catecholestrogens are mutagenic because they function as ROS-generating redox cycling agents (110–113). They are detoxified when methylated by catechol-*O*-methyltransferase (COMT) (114–116), which uses *S*-adenosylmethionine (SAMe) as donor for the methyl group, similarly as other methyltransferases (117). SAMe-dependent transmethylation reactions are subject to inhibition by the reaction product *S*-adenosylhomocysteine (118). The rate of catecholestrogen detoxification by COMT depends not only on the SAMe concentration in the cells, but also on the SAMe/*S*-adenosylhomocysteine concentration ratio. It must therefore be expected that the rate of catecholestrogen detoxification will be reduced by folate and vitamin B_12_ deficiencies, which reduce the rate of SAMe biosynthesis (117), and most likely also by vitamin B_6_ deficiency, which reduces the rate of homocysteine degradation by the trans-sulphuration pathway (117). The rate of SAMe biosynthesis can, moreover, also be reduced by methionine deficiency, which might be important in those parts of Africa where the dietary intake of sulphur amino acids is low, as in those humid parts of West Africa where cassava is used as a staple food (62). The expression of CYP1A1 and CYP1B1 is enhanced by several aryl hydrocarbon receptor (Ah receptor) agonists (119–121), including dioxins (122–124), PCBs (124–126), brominated flame retardants and some of their photochemical decomposition products or contaminants (124, 127–130), polycyclic aromatic hydrocarbons (PAHs) (124, 125, 131–133), and various herbicides and other pesticides (134–140). A synergistic interaction can therefore be expected, at least for all somatic cell lines expressing CYP1A1 and/or CYP1B1, between all these agents (including PAHs from tobacco smoke) and contraceptive pills containing human or equine estrogen. Cases where exposure to large quantities of Ah receptor agonists have been attended by high rates of foetal damage (as e.g. in Vietnam following exposure to the herbicide called Agent Orange (141)) should be studied very thoroughly in order to find out to what extent the causes are genetic (cf. 142) and to what extent one might deal with strictly teratogenic mechanisms (i.e. developmental disturbance without any harmful mutation being involved as a causal factor).

It was recently reported in a study from California that the risk of giving birth to autistic children was enhanced nearly by a factor of 2 [odds ratio (OR)=1.86; 95% confidence interval (CI), 1.04–3.45] among women who were living less than 309 m from a freeway at the time of delivery (143). Autism was also associated with residential proximity to a freeway during the third trimester (OR=2.22; CI, 1.16–4.42) (143). After adjustment for socioeconomic and sociodemographic characteristics, these associations were unchanged (143). Living near other major roads at birth was not associated with autism (143). While these observations certainly are consistent with the hypothesis that environmental pollution may play an important role in the etiology of autism, they can not tell if this is because of enhancement of the rate of mutations or because of some other, non-genetic mechanism.

It has also recently been reported that metabolic conditions (diabetes, hypertension, and obesity) during pregnancy are associated with enhanced likelihood of ASD and developmental delays (DD) in the child (144). All these metabolic disturbances were more prevalent among case mothers compared with controls (144). Collectively, they were associated with a higher likelihood of ASD and DD relative to controls (odds ratio: 1.61 [95% confidence interval: 1.10-2.37; odds ratio: 2.35 [95% confidence interval: 1.43–3.88], respectively) (144). Among ASD cases, children of women with diabetes had Mullen Scales of Early Learning (MSEL) expressive language scores 0.4 SD lower than children of mothers without MCs (P<0.01) (144). Among children without ASD, those exposed to any metabolic condition (diabetes, hypertension, or obesity) were found to score lower on all MSEL and Vineland Adaptive Behavior Scales (VABS) subscales and composites by at least 0.4 SD (P<0.01 for each subscale/composite) (144). The mechanisms explaining this have still not been adequately studied. However, it is known that the aldehyde methylglyoxal, which is mutagenic (145–147), is formed as an obligatory byproduct during glycolysis (148). It is therefore a reasonable speculation that the association between diabetes in the mother and enhanced risk of ASD in the child might partly be explained by a mutagenic effect of hyperglycemia on female germ cells and that this mutagenic effect partly might be mediated by enhanced glycolysis leading to enhanced production of methylglyoxal. Methylglyoxal is scavenged by two enzymes called glyoxalase-1 and glyoxalase-2 in a cyclic pathway, where reduced glutathione (GSH) is consumed in the glyoxalase-1 reaction and regenerated in the glyoxalase-2 reaction (149). It might thus be speculated that there possibly might be a synergistic interaction (depending on the *K*_M_ value of glyoxalase-1 for GSH) between hyperglycemia and GSH depletion (cf. 150) as causes of enhanced methylglyoxal-mediated DNA damage. In men it is possible that this mechanism might be much less important because of a biochemical labour division between the Sertoli cells and the germ cells (similarly as has been proposed for astroglia and nerve cells in the brain (151)), where the Sertoli cells take up glucose and convert it to lactate that is next exported to the germ cells, which use it as fuel for mitochondrial ATP production (152, 153). A strong association between obesity and male infertility has nevertheless been found, but it may probably in large measure be explained by other mechanisms, including endocrine disturbances (154, 155). The possibility can, of course, not be excluded that some of the same environmental or lifestyle factors that may lead to enhancement of the mutation rate in male germ cells (e.g. too much toxic heavy metals or a high dietary omega-6/omega-3 fatty acid ratio) also may play a role as causes of disturbances in appetite regulation or energy metabolism that may contribute to the development of obesity.

A large number of studies have addressed the question, whether there is an association between the age of the parents and the risk of getting an autistic child, and have also found an association between the age either of the mother or of the father and the risk of autism or other ASD in the child, but not always an age effect both for the mothers and the fathers (156–176). Although the data are not completely consistent regarding an effect of parental age both in men and women on the risk of autism in the child, the cumulative evidence for an association between parental age and the risk of autism in the children must nevertheless be considered very robust and strong. This is in turn highly consistent with the hypothesis that novel germline cell mutations both in men and women may be important in the etiology of autism. In one study it was found, however, that the age of the grandparents was also strongly associated with the risk of having an autistic child, apparently more so than the age of the parents themselves (169).

For women, similarly as for men, it must be expected that there will be a direct multiplicative interaction between enhanced rates per year of mutation in their germ cells and late reproduction (which is now very common in most, if not all industrial countries). If we wish to bring down the average germline mutation rate in large human populations, one of the most important practical steps should therefore be to encourage men and women to become parents while they are still very young and before they have finished their professional education. This might be practically difficult, but not impossible, and would be a small price to pay for the survival of our species. From a psychological point of view, it is of course not a bad thing either for men or women to help another to make children when they are at that age when the instinctive urge of doing so and the emotional reward both are at their strongest.

The Eigen error threshold is defined as the highest mutation rate per base pair that a population of any viral or cellular species can tolerate without progressive accumulation of errors in the genetic message faster than they can be removed by Darwinian selection processes (177, 178). This process is called mutational meltdown (179–181), and will sooner or later lead to total extinction of the population concerned. The Eigen error threshold may be calculated theoretically from plausible assumptions regarding the efficacy of selection mechanisms when the size of the genome (measured as the total number of DNA base pairs) is known. It is also possible to measure the natural mutation rates both in viruses and cellular organisms. Such studies have shown that the natural mutation rates of several viral, prokaryote and eukaryote species, from RNA viruses to *Homo sapiens*, are often so high that they are barely below the Eigen threshold (182–185). The reason is probably that the viruses or cellular organisms concerned live in a highly shifting world, and high mutation rates help them to cope faster through evolutionary (i.e. genetic) adaptation to changes in important environmental factors, such as temperature or the arrival of new pathogens. This situation leaves, however, no safety margin, if the mutation rate should be artificially enhanced.

The natural mutation rate in higher primates can be calculated by comparing the human and chimpanzee genomes, and has been found to be surprisingly high (182, 186–188), allowing no tolerance for further enhancement because of artificially induced mutations before the Eigen error threshold will be exceeded and the entire affected population will go extinct because of mutational meltdown. The actual germline mutation rate in human populations has not been well enough studied, but various forms of evidence (i.a. the rapid rise in the reported incidence of autism) suggest that the average mutation rate in large populations may now be much higher than the natural one. If this is correct, *Homo sapiens* may soon follow the dinosaurs, the sabre-toothed tigers and the mammoth, but by his own hands through a process of collective genetic suicide, unless this problem can be corrected before it is too late.

This threat, if real, should be considered as far more serious than any disease causing the death only of individual patients without affecting future generations and also far more serious, from a political point of view, than the problem of anthropogenic enhancement of the level of greenhouse gases in the atmosphere. No scientific manpower or economic resources should be spared in a global effort to avert this kind of catastrophe. But it may probably be very difficult to tackle since unnatural chemical mutagens (plus dietary deficiency conditions leading to enhancement of mutation rates) permeate our modern high-tech societies almost everywhere with modern medicine being no exception. It should, nevertheless, be considered the first and highest priority of the best biomedical scientists in the world, of research-funding agencies and of all medical doctors to try to stop the express train carrying all humankind as passengers on board before it arrives at the end station of our civilization.

Modern medicine may, however, itself be an important part of the problem because of far too much laxity in the control of how mutagenic drugs are used. It might therefore be difficult for medical doctors to persuade politicians and the general public to do what is needed in other sectors (e.g. agriculture) for reducing the rate of germline mutations unless they are willing to start with perhaps very wide-reaching and radical reforms in their own profession by stopping to use all such mutagenic drugs that are now used for treatment of non-lethal conditions (like anxiety, depression, common pains and fever) and try to substitute as much as possible mutagenic therapies with non-mutagenic ones even for the treatment of lethal diseases, such as AIDS, tuberculosis and cancer.

As an example of current laxity concerning the usage of mutagenic drugs might be mentioned paracetamol, which is still very widely used for reduction of common pains and fever more than 25 years after it was first reported to be mutagenic (9). It does not have any curative effect for any lethal disease, since it does not help to eradicate either viruses or bacteria, but only alleviates one of the symptoms commonly attending infectious diseases. Another example is the widespread use of nucleoside analogue reverse transcriptase inhibitors for treatment of AIDS. Most or all of these drugs have as an important side effect that they do not only inhibit the viral reverse transcriptase, but also DNA polymerase-*gamma* from the host (189). This enzyme is not only needed for DNA replication in the mitochondria (which may help to explain some of the commonly observed side effects of the drugs concerned (190, 191)), but also for all DNA repair processes there (192–194) – which means that the drugs concerned will enhance the mutation rate in the mitochondria of all cell types that are not completely shielded by lack of drug transporters in their plasma membrane or some other form of impermeable barrier. But it has never been convincingly demonstrated that either human oocytes or human male germ cells are effectively shielded against the drugs concerned.

To eradicate all of the important unnatural chemical mutagens all over the world (plus some natural ones being present at unnaturally high levels) and also all dietary deficiency conditions leading to enhanced rates of mutagenesis will most likely be far more difficult than to stabilize the level of greenhouse gases in the atmosphere. The only hope for persuading politicians and their voters to do what is needed is by collaboration between several of the best and most respected medical and other biological scientists in the world. However, to persuade the voters and the politicians to do what is needed to avoid utter catastrophe, it is also needed that biological scientists and religious leaders from all major religions collaborate, since the voice even of the most respected biomedical scientists in the world might easily be heard only by well-educated people (who have enough background knowledge about biology to understand what the scientist says and that it is indeed well founded), while the voices of respected religious leaders, especially if they might stand together regardless of which religion they represent, may go to the hearts of almost everybody regardless of whether they have much or very little education. A new international organization (similar to ‘International Physicians for the Prevention of Nuclear War’) might be needed to work with this challenge both at a scientific and political level. Its leader should be somebody who is not only a top-level biomedical scientist, but also well-known enough to the mass media and general public all over the world that his or her voice will be heard, while also at the same time not being too controversial for the religious leaders. ‘Friends of the human genome’ is proposed here as a possible name for that organization.
